# Learning deep features for dead and living breast cancer cell classification without staining

**DOI:** 10.1038/s41598-021-89895-w

**Published:** 2021-05-13

**Authors:** Gisela Pattarone, Laura Acion, Marina Simian, Roland Mertelsmann, Marie Follo, Emmanuel Iarussi

**Affiliations:** 1 Facultad de Farmacia y Bioquímica, Universidad de Buenos Aires, Buenos Aires, Argentina; 2 Faculty of Medicine, Albert Ludwigs University of Freiburg, Freiburg, Germany; 3 Instituto de Cálculo, Facultad de Ciencias Exactas y Naturales, Universidad de Buenos Aires, Buenos Aires, Argentina; 4 Instituto de Nanosistemas, Universidad Nacional de San Martín, San Martín, Argentina; 5 Dept. Medicine 1, Freiburg University Medical Center, Freiburg, Germany; 6 Universidad Tecnológica Nacional, Buenos Aires, Argentina; 7 Consejo Nacional de Investigaciones Científicas y Técnicas, Buenos Aires, Argentina

**Keywords:** Breast cancer, Machine learning

## Abstract

Automated cell classification in cancer biology is a challenging topic in computer vision and machine learning research. Breast cancer is the most common malignancy in women that usually involves phenotypically diverse populations of breast cancer cells and an heterogeneous stroma. In recent years, automated microscopy technologies are allowing the study of live cells over extended periods of time, simplifying the task of compiling large image databases. For instance, there have been several studies oriented towards building machine learning systems capable of automatically classifying images of different cell types (i.e. motor neurons, stem cells). In this work we were interested in classifying breast cancer cells as live or dead, based on a set of automatically retrieved morphological characteristics using image processing techniques. Our hypothesis is that live-dead classification can be performed without any staining and using only bright-field images as input. We tackled this problem using the JIMT-1 breast cancer cell line that grows as an adherent monolayer. First, a vast image set composed by JIMT-1 human breast cancer cells that had been exposed to a chemotherapeutic drug treatment (doxorubicin and paclitaxel) or vehicle control was compiled. Next, several classifiers were trained based on well-known convolutional neural networks (CNN) backbones to perform supervised classification using labels obtained from fluorescence microscopy images associated with each bright-field image. Model performances were evaluated and compared on a large number of bright-field images. The best model reached an AUC = 0.941 for classifying breast cancer cells without treatment. Furthermore, it reached AUC = 0.978 when classifying breast cancer cells under drug treatment. Our results highlight the potential of machine learning and computational image analysis to build new diagnosis tools that benefit the biomedical field by reducing cost, time, and stimulating work reproducibility. More importantly, we analyzed the way our classifiers clusterize bright-field images in the learned high-dimensional embedding and linked these groups to salient visual characteristics in live-dead cell biology observed by trained experts.

## Introduction

Breast cancer is the most frequently diagnosed malignancy in women worldwide; one out of eight women are expected to develop breast cancer at some point in their lifetime^1^. As a disease, it involves biologically diverse subtypes with high intratumor heterogeneity that determine different pathological characteristics and have different clinical implications. Understanding the intricacy of the molecular cross-talk within the cell death pathway highlights the need for developing methods to characterize the morphological cell response to therapy with anticancer drugs. The emergence of automatic microscopes made it possible to develop large datasets of live fluorescence images and single cell analysis, and more recently, these data started to be massively studied by means of computational tools. Some efforts are focused on developing image processing programs able to identify cells and separate them from the extracellular matrix, performing segmentation and tracking cells using contrast fluorescence^2^. More recent efforts are based on automatic classification of images using deep learning techniques^3, 4^, a form of automatic learning^5, 6^ enabling improved data analysis for high-throughput microscopy^7, 8^. For example, deep convolutional neural networks have been trained^9^ with labeled images from different cell types like motor neurons, stem cells, and Jurkat cells^10^. In order to label each cell, Hoechst and DAPI have been used to identify nuclear areas, CellMask to highlight plasma membranes and Propidium Iodide to spot cells with compromised membranes. These automatic methods were able to make accurate pixel predictions of the location and intensity of the different structures represented by the fluorescence. More recently, machine learning classifiers were trained to perform stain-free hierarchical classification of human white blood cells in flow cytometry images^11^. Similar methods have been used to distinguish dead from living microalgae Chlorella vulgaris with features extracted from individual cells^12^. In both cases, the acquisition technique isolates cells, simplifying segmentation and labeling tasks in the image preprocessing step. In the context of cancer cell growth, this type of isolation is difficult to achieve, making it necessary to use techniques which can aggregate image information and automatically extract features for classification. Other innovative biological applications related to automated image processing methods are morphological classification of hematopoietic cells, pluripotent stem cells^13^ and 3D cell boundary and nuclear segmentation^14^.

Empowered by recent advances in image processing and deep learning, in this work we were interested in the study of morphological characteristics showing death signs in breast cancer cells. Particularly, in the context of live cell fluorescence, the live-dead labeling method has many limitations like low contrast or differences in pixel intensities, resulting in heterogeneous staining for individual cells and requiring a final human-assisted cell segmentation. Additionally, fluorescent stains are expensive and usually several stains are required to precisely identify a cell^11^. Fluorescence-free cell classification could potentially offer substantial improvements in detection specificity, sensitivity, and accuracy for physiological and pathological cell condition diagnosis. Furthermore, the cells could remain in their regular culture conditions without any intervention. Our purpose is to evaluate the potential of automatically classifying cancer cells as live or dead without staining, using only bright-field images as input.

**Figure 1:**
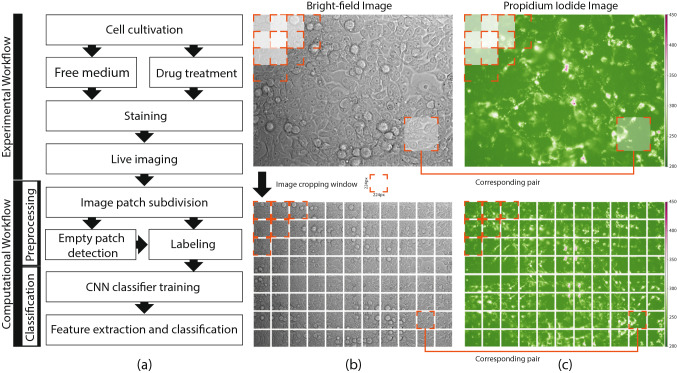
(**a**) Experimental and computational steps in our automated cell classification pipeline (diagram created using Adobe Illustrator CC 2019 https://www.adobe.com/products/illustrator.html). (**b,c**) Top: bright-field and corresponding fluorescence images resulting from the imaging step (experimental workflow). High fluorescence values (white and red areas) indicate cell death. Bottom: as a post-process, images are cropped into 224 $\times$ 224 px patches and paired with their corresponding fluorescence patch. Notice cropping overlaps contiguous patches (horizontal and vertical) in order to augment the number of images (images rendered using Matplotlib 3.3.3 https://matplotlib.org/).

First, we present a new massive dataset of breast cancer cell images of the JIMT-1 breast cancer cell line^15^. We studied cellular growth before and after the introduction of in vitro drugs treatments with Doxorubicin and Paclitaxel. After characterizing the biological behavior within chambered coverslips, each image was split into smaller patches containing a very limited amount of cells and properly tagged as live or dead using the information available in the form of fluorescence images from calcein and propidium iodide. To our knowledge, no other dataset of labeled JIMT-1 cell images has been compiled and publicly released before. We then used this dataset to train deep CNN models for cell image classification. These trained classifiers learned to label cancer cells as live or dead without staining and using only bright-field images as input. A diagram of the presented workflow is shown in Fig. 1a. We additionally studied the learned embeddings and identified clusters of images with similar visual cues which are often associated with living and dead cells. We believe our results could be helpful as a diagnostic and complementary tool for cancer and normal cell biology, allowing a better understanding of the capabilities of image-based automatic classification. Furthermore, we foresee potential applications in the pharmaceutical field, as automatic live/dead cell classification in preclinical trials for drug tests is of high interest, complementing the information related to pharmacokinetics and pharmacodynamics characteristics of new anti-cancer drugs development.

## Results

### Cell preparation and image acquisition

To ensure a biologically representative set of breast cancer cell images in our dataset, we first analyzed and characterized the development of JIMT-1 within the Ibidi chamber slides. JIMT-1 cells are positive for cytokeratins 5/14 and 8/18, are estrogen and progesterone receptor negative, and overexpress HER2 as a consequence of HER2 amplification. JIMT-1 cells are classified as basal-like and represent the subgroup that occasionally carry HER2 amplifications. JIMT-1 cells act like a triple negative subtype breast cancer given their lack of response to trastuzumab^15^. To induce JIMT-1 cell death we designed a treatment scheme consisting of a 4 h exposure to doxorubicin followed by 24 h of paclitaxel. In order to capture the images, we performed live fluorescence imaging using a live-dead cell imaging kit of cells cultured in chambered coverslips with 8 independent wells and a non-removable polymer coverslip-bottom, over extended time periods. This setup has high optical quality, with a total growth area per well of 1.0 cm$^{2}$, tolerates live fluorescence, and allows the tracking of breast cancer cells during a maximum of five days. We constructed a biologically representative dataset of breast cancer cells grown in culture medium supplemented with the sequential treatment of doxorubicin followed by paclitaxel. After cultivation and drug treatment, we measured the effect of the therapeutic agents on the percentage area shown by calcein and propidium fluorescence. Both were studied in comparison with a control sample. The area of activity of the PI fluorescence was higher in comparison to control. Simultaneously, the calcein percentage area was lower at the end of the treatment. Both facts combined showed that the treatment with drugs was effective in inducing cell death and ensured that our image dataset contained both cells states, live and dead. We compiled 964 raw images into a dataset we named Doxo/Paclitaxel. We additionally collected 339 raw images from the cell growth and death process occurring spontaneously (without therapy) during the same time period and named it No treatment. In both datasets, each bright-field image has a corresponding fluorescence PI image indicating cell death (Fig. 1b,c, top).

### Image pre-processing

**Figure 2:**
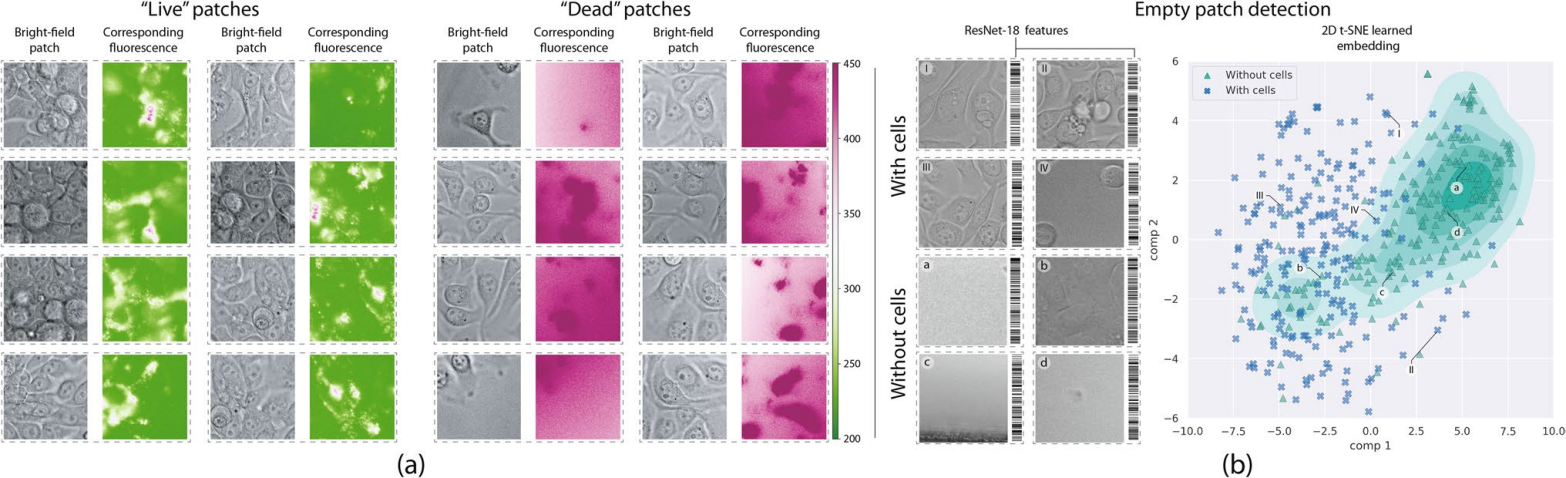
(**a**) Bright-field and corresponding propidium iodide fluorescence images. The columns under Live patches (green) show images with mostly live cells. The columns under Dead patches (red) present images with mostly dead cells in our dataset (images rendered using Matplotlib 3.3.3 https://matplotlib.org/). (**b**) Left, samples of empty and non-empty patches with the associated barcode visualization of the 512-dimensional feature vector from ResNet-18 last convolutional output per image. Right, dimensionality reduction visualization of patch features and the high-dimensional decision function (green level-sets) learned by the SVM. Notice how empty patches mostly lie inside the highlighted region (images rendered using Seaborn 0.11.1 https://seaborn.pydata.org/ and Matplotlib 3.3.3 https://matplotlib.org/).

We curated the raw images to be suitable for training automatic classifiers. We identified several problems with the raw images that we solved individually in order to prepare the final image set. The first issue relates closely to the image size. Raw images cover large areas of the Ibidi device with a resolution of 1344 $\times$ 1024 pixels, and often the associated PI fluorescence strongly varies across it. This represents a problem in our setup, since a single label indicating live or dead must be assigned to each image to train the classifier. Therefore, we decided to partition raw images into smaller patches (Fig. 1b,c, bottom). By cropping smaller areas, we increased the reliability of the labels for each patch, since neighboring cells often have the same state. However, setting a proper granularity for this operation is not trivial. On the one hand, individually labeling each cell could lead to very accurate labels, but the topology of cell growth in the device makes it difficult to automatically isolate cells. On the other hand, cropping large areas could lead to overlapping labels, with interfering residual fluorescence from neighboring patches. Despite the fact that PI has the characteristic of only entering the cell when its membrane is compromised, we noticed the fluorescence spectrum emissions are not uniform and may overlap or even occupy more than one cell diameter. We therefore found a compromise between these two options by using a fixed size sliding cropping window. Conveniently, we cropped 224-pixel wide square patches, a standard size that facilitates the use of widespread CNN backbones (see “Classifiers training”). In our datasets, each bright-field cropped patch has a corresponding cropped fluorescence image (Fig. 2a).

After cropping, we noticed many image patches did not capture any cells. This is especially common in data coming from the first culture days, where a uniform distribution is not yet achieved. When training automatic classifiers, empty images can decrease network performance because no real feature extraction process occurs without cells in the image. We therefore implemented a mechanism to easily detect and discard empty patches. First, we manually labeled a subset of 226 bright-field patches that didn’t contain any cells or unsuitable data, such as out of focus images, and 226 patches containing properly captured cells (Fig. 2b, left). For each of these images, we computed a 512-feature vector by taking the output of the last convolutional layer from a pretrained ResNet-18 on ImageNet^16^. We did not perform any fine-tuning of this network using our images. Reusing features from other CNN-learned representations is a common practice in anomaly and outlier detection^17, 18^. The no-cell dataset contained patches from both: no treatment and Doxo/Paclitaxel data partitions. We then trained a support vector machine (SVM)^19^ to perform outlier detection using ResNet-18 features. The trained model learned to detect most of the empty images (f1-score = 0.833). Figure 2 (**b**) presents a 2D t-distributed stochastic neighbor embedding (t-SNE)^20^ visualization of the learned high-dimensional decision function when classifying image patches as with or without cells. After cropping and filtering empty patches, the No treatment set contains 21,848 images and the Doxo/Paclitaxel set contains 56,632 images.

**Figure 3:**
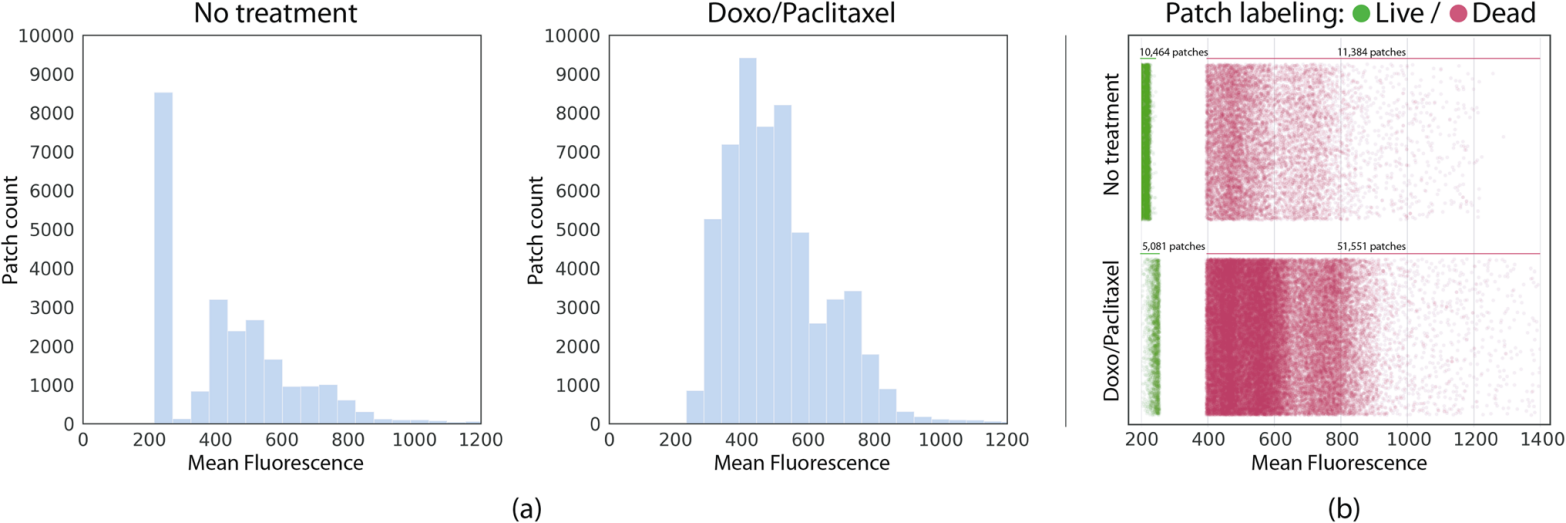
(**a**) Histograms of mean fluorescence per patch on each dataset. (**b**) Scatter diagram showing the samples distribution after the automatic patch labeling based on their mean fluorescence. Due to treatment, Doxo/Paclitaxel has significantly less live patches (images rendered using Seaborn 0.11.1 https://seaborn.pydata.org/).

Once most empty images were removed from the datasets, we prepared them for supervised training, that requires a single binary label indicating whether the image represents live or dead cells. We therefore averaged the fluorescence values and set up a threshold splitting the image in two non-overlapping sets: a set labelled as containing live cells and another containing dead cells. We found the threshold for defining each set by computing histograms of the mean fluorescence intensities for the No treatment and Doxo/Paclitaxel datasets (Fig. 3a). Choosing a very high threshold (high fluorescence values) would assure more certainty for image patches labeled as dead, but it would end up labeling as alive many images that are far from the low fluorescence values indicating live signs. Conversely, the opposite effect would be observed if choosing a very low threshold. We solved this issue by fitting a Gaussian mixture model^21^ to the No treatment distribution of means which contained the most balanced number of live and dead cells. We identified two main clusters in data: one with very low fluorescence values containing mostly live cells ( ${\overline{x}}_{live}$ = 224.51, $s_{live}$ = 34.46), and another one with high fluorescence values containing mostly dead cells (${\overline{x}}_{dead}$ = 550.44, $s_{dead}$ = 153.55). We used this model to label as live all images with mean fluorescence lower than ${\overline{x}}_{live} + s_{live}$ = 258.97, and as dead all the images with mean fluorescence above ${\overline{x}}_{dead} - s_{dead}$ = 396.89. Patches in the range $({\overline{x}}_{live} + s_{live}, {\overline{x}}_{dead} - s_{dead})$ were discarded. We applied the same threshold to both, No treatment and Doxo/Paclitaxel datasets (Fig. 3b). Only very low fluorescence values are considered as containing live cells. Table 1 summarizes the number of images included in each pre-processing step and available in this repository: https://github.com/emmanueliarussi/live-dead-JIMT-1.

**Table 1:** Dataset summary.

	Raw images	Cropped images	Total live	Total dead	Train live	Train dead	Train total	Valid. live	Valid. dead	Valid. total	Test live	Test dead	Test total
No treatment	339	21,848	10,464	11,384	8680	9480	18,160	891	925	1816	979	893	1872
Doxo/paclitaxel	964	56,632	5081	51,551	4195	42,966	47,161	437	4314	4751	449	4271	4720

The first row corresponds to raw, full resolution images (1344 $\times$ 1024 pixels). The number of cropped images are reported after empty patch filtering. The classification classes are unbalanced, particularly for the Doxo/Paclitaxel data.

### Classifiers training

We trained three different CNN backbone architectures to perform binary live-dead classification using the curated cell image dataset: ResNET-18^22^, SqueezeNET^23^, and Inception-v3^24^. Each network architecture was trained twice using a cross entropy loss function and the No treatment and Doxo/Paclitaxel dataset partitions. Three splits for each dataset were constructed to allow training and subsequent evaluation tasks (Table 1). Approximately 80% of the images were used for training, 10% for validation and 10% for testing, as suggested in the literature^25^. Since each cropped image patch was tagged with an identifier corresponding to the ID of the raw image from which it came, we were able to avoid patches from the same raw image to belong to more than one partition simultaneously. In other words, there are no overlapping images among training, validation, and testing partitions since we carefully selected patches from different raw images for each set.

**Figure 4:**
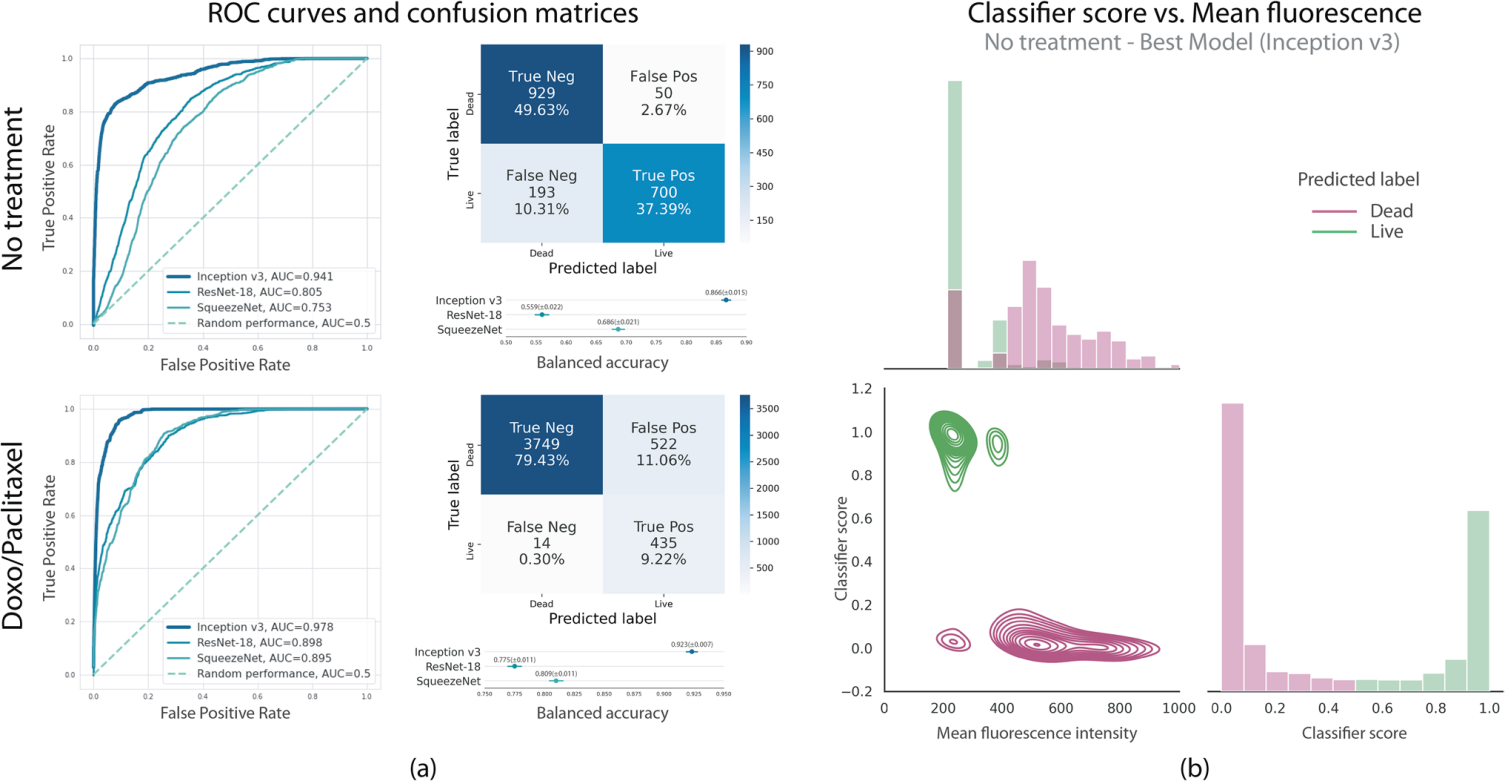
(**a**) ROC curves showing the classification performance over the testing datasets for each CNN architecture. The Inception-v3 model outperforms ResNET and SqueezeNET (plotted in Matplotlib 3.3.3 https://matplotlib.org/). (**b**) Mean fluorescence vs. classifier-score analysis for the model that performed best over No treatment data (Inception-v3). Higher mean fluorescence intensities tend to cluster together for lower classification scores used to label dead cells. Simultaneously, lower mean fluorescence intensities are grouped near higher classification scores that signal live cells (images rendered using Seaborn 0.11.1 https://seaborn.pydata.org/).

A common problem when training classifiers is their sensitivity to class imbalance^26^. Therefore, to compensate for the strong imbalance in our dataset, we sampled images by means of a weighted random sampler with replacement. Weights were computed as the inverse of the sample count for each class. Additionally, data were augmented by random 90-degree rotations and vertical/horizontal flipping of each image. This type of data augmentation leads to better generalization performance^11^. We empirically found that fine-tuning network weights pre trained on Image-NET^27^ performed significantly better than training from a random initialization. Therefore, we adopted a transfer-learning approach for all the reported results. Training hyperparameters were adjusted based on the network performances over the validation set. More training details can be found in the Methods section.

After training, each model was validated using non-augmented instances from the validation set. In order to evaluate and compare the performances of the trained classifiers, we relied on several metrics. In particular, we computed the balanced accuracy, which is defined as the average of the recall obtained on each class^28, 29^. This metric is well-suited for our setup, since it does not favor a classifier that exploits class imbalance by biasing toward the majority class. Together with the balanced accuracy, we computed confusion matrices and pairwise relationships between mean fluorescence and the classifier score. Figure 4a summarizes the performance of the trained classifiers. Overall, the three models outperformed random performance for both datasets and were able to automatically extract relevant image features in order to classify JIMT-1 cell images as living or dead. Inception-v3 was the best performant model, with over 85% accuracy over both testing datasets, No treatment balanced accuracy = 0.866 (95% CI = [0.851, 0.881]), AUC = 0.941; Doxo/Paclitaxel balanced accuracy = 0.923 (95% CI = [0.916, 0.930]), AUC = 0.978. Confusion matrices and ROC curves in Fig. 4 further illustrate the classifiers’ performance. Furthermore, we computed the correlations between the mean values of PI and the classification score obtained for each image in the testing set to explore the association between classification and fluorescence images. A significative inverse Pearson correlation was found in both training scenarios, No treatment: r = − 0.705 (p = 0.024) and Doxo/Paclitaxel: r = − 0.281 (p = 0.025), indicating the scores are correlated to the fluorescence levels, a relationship that could be explored in future work in order to predict fluorescence images from bright-fields (Fig. 4b).

### Visualizing learned features

In line with previous work^10, 11, 30, 31^, we took advantage of well-known visualization techniques in order to gain further insight into the classifiers’ automatically learned space to uncover their biological meaning. We now show a series of complementary visual analytics and link the observed common patterns to salient visual characteristics in live-dead cell biology observed by trained experts and reported in literature. The presented feature space visualizations and the class activation maps are intended to complement the quantitative study, providing ‘visual explanations’ for decisions from the CNN models. These visualization techniques are developed to reveal how these models localize discriminative regions for an input image. Such understanding provided insights into the model to our biomedical specialist co-authors (Drs. Pattarone and Simian), but is not intended to be used right away in a lab environment.

**Figure 5:**
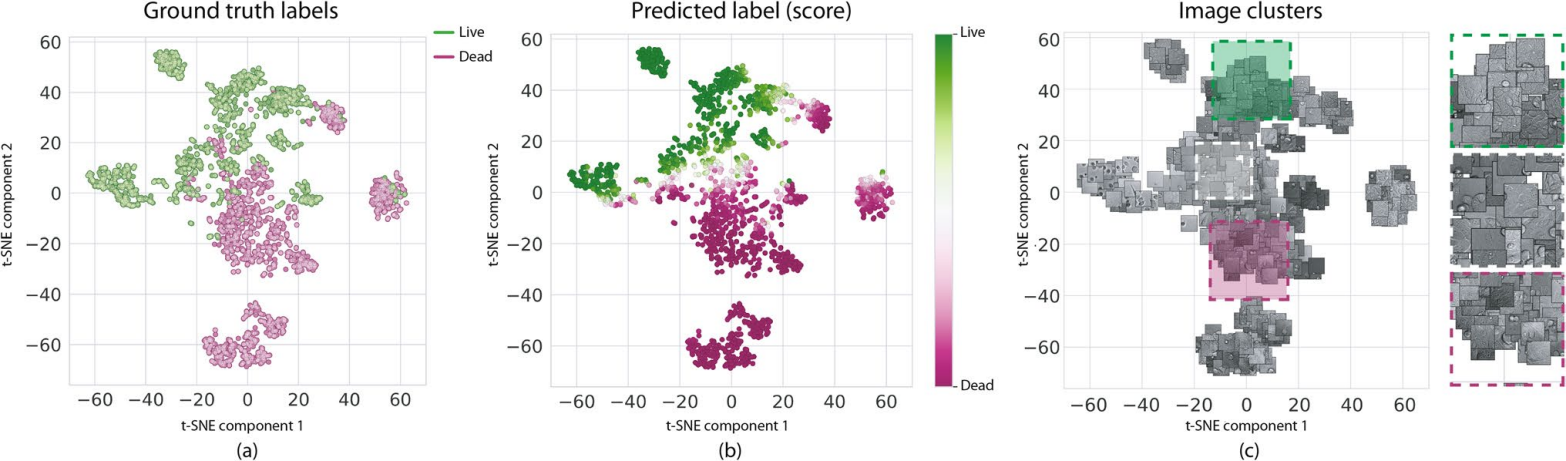
Visualization of the Inception-v3 learned feature space for our No treatment testing dataset. The 2048-dimension features were projected to a 2D space using t-SNE, and colored according to ground truth labels (**a**), and predicted labels (**b**). Cells with the same state tend to cluster together. Visual inspection of the images in each cluster further reveals the shared characteristics within each group. All images were rendered using Seaborn 0.11.1 https://seaborn.pydata.org/ and Matplotlib 3.3.3 https://matplotlib.org/.

In particular, we applied a nonlinear dimensionality reduction technique suited for embedding high-dimensional data into a low-dimensional space, namely t-SNE^20^, which preserves local structures of the high-dimensional input space. The learned features of a CNN are encoded by the intermediate activation after the last convolutional layer of the network. Therefore, given an input image which is fed to the CNN to perform classification, we extract the activation pattern of the last layer before classification. This high-dimensional vector becomes a signature of the input image. Scatter plots in Fig. 5a,b illustrate the emerging clusters after projecting the 2048-dimensional features of Inception-v3 into two components for all testing samples. To further understand each cluster, we also show a version of the scatter plot where each dot is replaced by the corresponding bright-field image thumbnail (Fig. 5c). This enhanced visualization reveals that groups of cell images with similar visual characteristics tend to cluster together in the learned feature space. This visualization of the feature space learned by the classifiers also provided a visual validation of the classification confusion occurring between live and dead cells. We found that the boundary between main live and dead clusters (white dots in Fig. 5b) correspond to images in which a mixture of live and dead cells appear.

**Figure 6:**
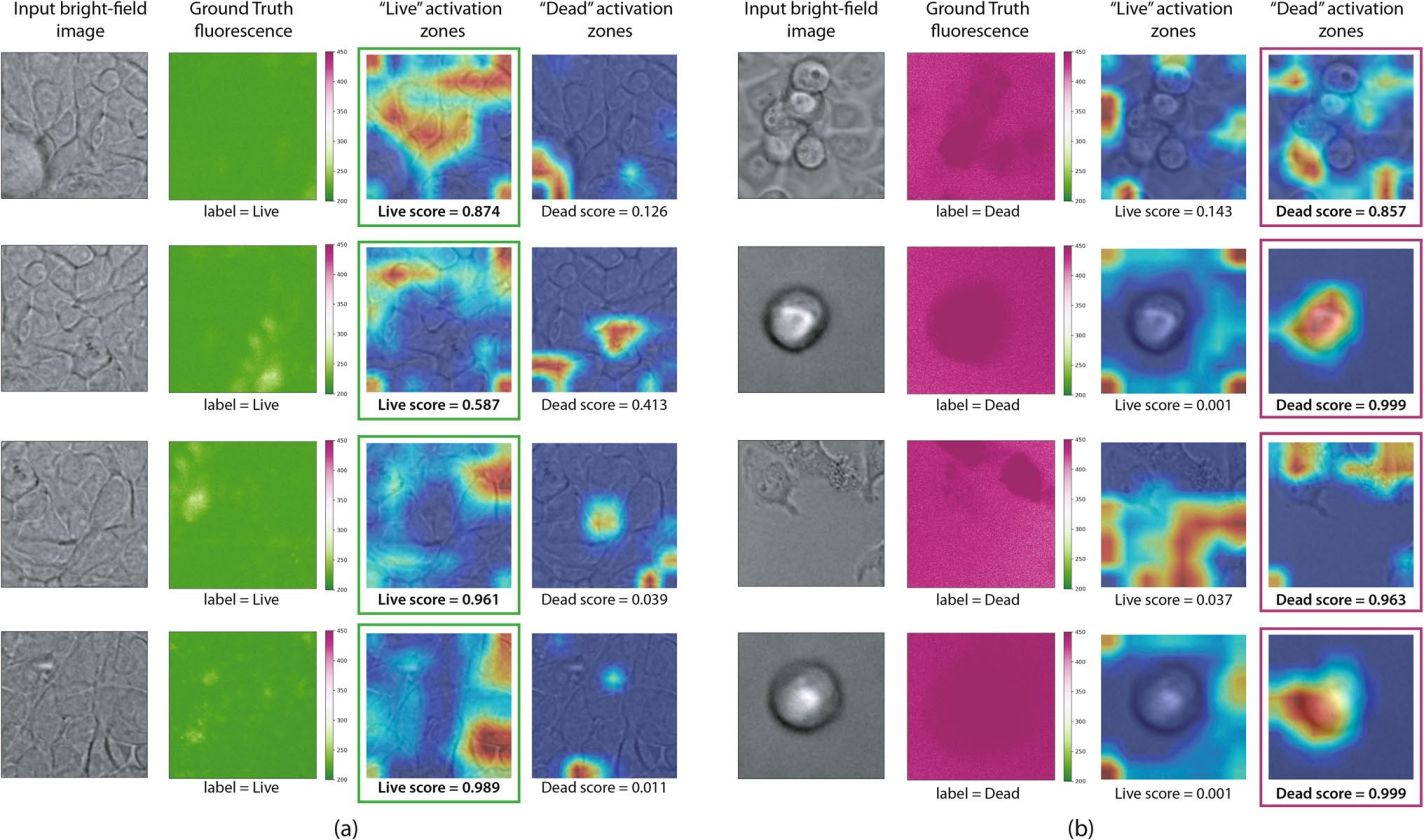
Bright-field testing patches paired with gradient-weighted class activation mapping (Grad-CAM) visualizations for the Inception-v3 model. Live patches are shown on the left (**a**), and dead on the right (**b**). The activation maps show which zones of the input bright-field are triggering the classifier response. These maps can be computed for both labels and help to identify zones in the input images activating a live or a dead response from the convolutional neural network. The maps also have an associated score, indicating the probability of each label, which determines the final classifier response. All images were rendered using Grad-CAM 1.0 https://github.com/jacobgil/pytorch-grad-cam and Matplotlib 3.3.3 https://matplotlib.org/.

Complementary, we investigated the relation between input bright-field images and the produced outcomes by means of the gradient-weighted class activation mapping (Grad-CAM)^32^. This visualization technique uses the class-specific gradient information flowing into the final convolutional layer of a CNN to produce a coarse localization map of the important regions in the image which triggered the classifier output. These regions can be visualized by means of a heatmap (Fig. 6).

Overall, in the case of living and untreated cells, morphology looks as expected, with the presence of an uncompromised membrane, organelles, nuclei, and nucleolus (Fig. 6a). The membrane can be often seen clearly without any special enhancement (green fluorescence rows in Fig. 6). This integrity of the cell membrane is necessary to keep the position of its organelles, mainly rough endoplasmic reticulum and golgi apparatus. Cells in this group have a mostly uniform gray color, scattered by very tiny dark circles, possibly corresponding to the cell nuclei. These are expected morphological characteristics of a cell that remains active and where its chromatin remains partially in the form of a nucleolus and that is decomposed and used according to the needs of the biological machinery. Biological aspects of dead cells are different. It can be seen in patches containing stained and classified as dead cells (red fluorescence rows in Fig. 6b), that the compromised membrane appears more as a blurred dark halo. This is expected since the PI staining enters the cell only when the cell membrane has been compromised and binds to DNA by intercalating between the bases with little or no sequence preference. It can be generally observed that the harmonic disposition evidenced as a smooth gray of the organelles is lost, probably due to a contraction of the cytoplasm that occurs in the processes of cell death. The cell death process leads to a series of intracellular events, regulated, and coordinated by the activation of different enzymes that perform proteolysis cascades and controlled destruction of organelles and genetic material. The final phase of this process is evident inside the cells classified as dead. The circular genetic material known as nucleolus is not evident, but rather there is a deletion of it as can be clearly noted in cells identified as dead. On the contrary, cells identified as live maintain the central dark gray nucleoli. Differences in cell death images in the two groups, No treatment and Doxo/Paclitaxel datasets, can be seen in the process of contraction of the cytoplasm and DNA degradation. The pharmacological effect of Doxorubicin on the cancer cells is induced by intercalation into DNA and disruption of topoisomerase-II-mediated DNA repair and generation of free radicals that damage cellular membranes, DNA, and proteins^33^. This is supplemented by the effect of Paclitaxel on tubulin that polymerizes into small tubes called microtubules, which are responsible for mitosis, cell movements, preservation of cell shape, as well as the intracellular trafficking of organelles and macromolecules. Paclitaxel stabilizes microtubules and reduces their dynamicity, promoting mitotic halt and cell death^34^. Both pharmacological effects can be visualized in the cytoplasms that present a kind of effacement and bright spot in the brightfield image, without evidence of destruction of organelles and genetic material.

## Discussion

All evaluated network architectures were able to autonomously extract relevant information from bright-field imagery in order to perform live-dead classification. This automatic feature extraction can be improved in future work, by combining it with cell characteristics i.e. cell diameter, area, and radius, similar to the work of Reimann et al.^12^. The mixture of learned and engineered features can improve performance as well as interpretability of the classifier behaviour. In order to push further in this hybrid direction there is a need for more robust methods able to individualize and segment cells growing as an adherent monolayer. At the beginning of this project, we explored the alternative of segmenting and labeling each cell individually before classification, but the extremely irregular cellular contours and the occasional overlap among them made this approach inapplicable. We believe the work of Lugagne et al.^35^ highlights the next steps to overcome these issues.

The curated image data was of paramount importance for the achieved performances of the classifiers. In general, the lack of large image datasets greatly hampers the applicability of deep learning techniques. Even if our dataset was big enough to learn and generalize to unseen samples, we believe a larger effort in building bigger and more diverse datasets is still necessary. For example, all our images come from a single capture device, which could limit the applicability of the trained models to images from a different acquisition setup. We also worked on a single cell line and stain. More data will definitely contribute to make these tools widely available across the scientific and medical community. Future work should consider compiling images in a variety of capture scenarios.

Another interesting research direction is to study how the applied techniques for providing visual explanations can complement classification tasks in the laboratory. A first step in this direction could be to conduct a comparative study using living and dead regions segmentations from the validation images and their corresponding activation maps.

Automatic cell classification is a very challenging and interdisciplinary problem, involving simultaneous efforts from computer vision, machine learning and biomedical research. In the context of human breast cancer, machine learning can bring new tools to support diagnosis that benefit the biomedical field by reducing cost and time. In this work we investigated the applicability of deep learning techniques to stain-free live-dead breast cancer cell classification from bright-field images. Since our aim was that others may reuse our findings and data, we used open-source Python packages and we made freely available our image dataset online.

## Methods

### Experimental methods

#### Cell culture

JIMT-1 cells ATCC 589 (DSMZ) were cultured in complete DMEM medium (Gibco), supplement with fetal calf serum heat-inactivated (FBS) 10% (w/v) (Gibco), l-glutamine 2 mmol L 1 (Gibco), penicillin 100 units mL $^{-1}$, streptomycin 100 g mL (Gibco) at 37 $^{\circ }$C in an incubator with 5% CO2. Cells were resuspended with trypsin 0.50 mg mL $^{-1}$ and EDTA-4Na 0.2 mg mL $^{-1}$1 (Gibco), and incubated at 37 $^{\circ }$C for 3 min. Trypsin was inactivated with FBS and cells were washed with phosphate buffer solution (PBS) (NaH2PO4 50 mmol L $^{-1}$ , NaCl 300 mmol L $^{-1}$ , (pH 7.6) and centrifuged at 1200 rpm for 5 min. Finally, the cells were resuspended in the same complete DMEM medium. We use the 8-well slide (Ibidi GmbH) and 12,000 cells per well were used to perform culture assays according to the manufacturer’s protocol.

#### Cell viability staining

We used the live-dead cell imaging kit (Sigma) to evaluate cell viability in the Ibidi chip. The cells were loaded into the Ibidi devices and cell viability was evaluated at third, fourth, and fifth days; we PBS to wash the culture chambers in the models for 1–3 min. Then, the cells were incubated with the live-dead cell imaging kit for 15–30 min at 37 $^{\circ }$C. Next, we used PBS again to wash out the reagent for 3–5 min and observed the culture chambers under a fluorescent microscope.

#### Autophagy and apoptosis activity staining

We used the autophagy cell imaging kit (CYTO-ID) and caspase-3 and-7 cell imaging kit (Invitrogen). In both assays performed separately, the cells are stained green. The procedure with negative and positive controls were performed as recommended by the manufacturers’ instructions (Enzo ENZ-51031-K200) 32.

#### Doxorubicin and paclitaxel schematic treatment

For the drug schematic tests, the effects of paclitaxel (Sigma Aldrich) and doxorubicin (Sigma Aldrich) combined were studied (Holmes 1996). First, JIMT-1, were loaded into the Ibidi chips, as described previously, and 24 h later when the cells were adherent, the medium was replaced with fresh culture medium supplemented with 0.01 M doxorubicin (DOX). Then, after 4 h it was subsequently replaced with a fresh medium containing 0.001 M paclitaxel (PAX) for 24 h. Live imaging and biological characterization with different staining as described before was performed for the whole experiment.

#### Microscopy

ell images were captured using the Olympus ScanR microscope. The images collected for the dataset were taken in each biological step related to cellular growth and the use of different chemotherapeutic agents and drug schemes. A 20$\times$ magnification was used, according to this each image has the dimension of 433 $\times$ 330 m, with a conversion factor 0.32250 m/pixel, and a final pixel per image 16 bit of 1346 $\times$ 1024 pixels. Each brightfield image taken by the microscope was triplicated in the same position by different filters chosen to show the biological structure labeled with the correspondent fluorescence. For the Höechst filter we used an excitation filter of 377/50 with an emission filter of 437–475 nm, for the propidium iodide filter we used an excitation filter of 575/25 with an emission filter of 600–662 nm, and for autophagy and caspase we used an excitation filter of 494/20 with and emission filter of 510–552 nm.

### Computational methods

#### Dataset construction

We converted the raw 16 bit microscope images to an unsigned 8 bit type (both bright-field and fluorescence images). Pre-computations were implemented in Python using OpenCV (Open Source Computer Vision Library) framework, an open source computer vision and machine learning software library.

#### Neural networks

The network architectures and training were implemented in Python using PyTorch Framework^36^ and the aforementioned pre-trained models. We used the same hyperparameters for all network architectures and training scenarios: learning rate lr = 1e−5, batch size bz = 4, epochs e = 30. We optimized our objective function by means of the Adam, a state of the art adaptive learning rate optimizer implemented in PyTorch (b0 = 0.5; b0 = 0.999), with weight decay wd = 1e−5.

#### Equipment

A notebook was used for the creation of the dataset. Training of the CNN was performed on an Intel Xeon server equipped with two Graphics Processing Unit (GPU) Nvidia Titan Xp and 32 Gb of RAM.

## Data Availability

The image dataset and further resources are available in the public github repository: https://github.com/emmanueliarussi/live-dead-JIMT-1
