# Author Correction: Learning deep features for dead and living breast cancer cell classification without staining

**DOI:** 10.1038/s41598-021-99144-9

**Published:** 2021-09-24

**Authors:** Gisela Pattarone, Laura Acion, Marina Simian, Roland Mertelsmann, Marie Follo, Emmanuel Iarussi

**Affiliations:** 1 Facultad de Farmacia y Bioquímica, Universidad de Buenos Aires, Buenos Aires, Argentina; 2 Faculty of Medicine, Albert Ludwigs University of Freiburg, Freiburg, Germany; 3 Instituto de Cálculo, Facultad de Ciencias Exactas y Naturales, Universidad de Buenos Aires, Buenos Aires, Argentina; 4 Instituto de Nanosistemas, Universidad Nacional de San Martín, San Martín, Argentina; 5 Dept. Medicine 1, Freiburg University Medical Center, Freiburg, Germany; 6 Universidad Tecnológica Nacional, Buenos Aires, Argentina; 7 Consejo Nacional de Investigaciones Científicas y Técnicas, Buenos Aires, Argentina

Correction to: *Scientific Reports* 10.1038/s41598-021-89895-w, published online 13 May 2021

Roland Mertelsmann and Marie Follo were omitted from the author list in the original version of this Article.

The Author Contributions section now reads:

“G.P. conceived the lab experiments and dataset, G.P. and E.I. conducted the computational pipeline. The laboratory experiments were performed in M. F`s. laboratory under her supervision and conceived and guided by R.M.. G.P., L.A., M.S. and E.I. analysed the results, wrote and reviewed the main manuscript text.”

In addition, the Acknowledgements section now reads:

“This study was supported by Agencia Nacional de Promoción Científica y Tecnológica, Argentina, PICT 2018-04517, Préstamo BID-PICT 2016-0222 and BID-PICT 2018-01582, donations from the Federico Deutsch Jack Yael Foundation, the Banchero Family and Grupo Día to M.S, PID UTN 2018 (SIUTNBA0005139), PID UTN 2019 (SIUTNBA0005534), and NVIDIA GPU hardware Grant that supported this research with the donation of two Titan Xp graphic cards. The Laboratory experiments were supported by the Biothera-Roland Mertelsmann Foundation and the Freiburg University Medical Center.”

The original Article has been corrected.

